# Editorial: Unveiling biomarkers and mechanisms in the tumor-immune nexus

**DOI:** 10.3389/fimmu.2025.1581492

**Published:** 2025-03-05

**Authors:** Chenfeng Ma, Wantao Wu, Pengpeng Zhang, Jiaheng Xie

**Affiliations:** ^1^ Department of Neurosurgery, The First Affiliated Hospital of Nanjing Medical University, Jiangsu Province Hospital, Nanjing, Jiangsu, China; ^2^ Department of Thyroid and Breast Surgery, The Second Affiliated Hospital, Chongqing Medical University, Chongqing, China; ^3^ Department of Lung Cancer Surgery, Tianjin Medical University Cancer Institute and Hospital, Tianjin, China; ^4^ Department of Plastic Surgery, Xiangya Hospital, Central South University, Changsha, China

**Keywords:** immunotherapy response biomarkers, tumor-immune interactions, immune evasion mechanisms, tumor microenvironment, next-generation sequencing technologies

## Introduction

Cancer is a multifaceted disease driven by genetic mutations, epigenetic changes, and the evolving interaction with the immune microenvironment (1). Tumor cells often evolve mechanisms to evade immune detection and promote immune suppression (2). Despite the recent breakthroughs in cancer immunotherapy, the identification of novel biomarkers and mechanisms that can predict therapeutic outcomes and provide more targeted treatments remains a significant challenge (3).

The tumor-immune nexus encapsulates the interactions between tumor cells and various immune cells, including T-cells, macrophages, dendritic cells, and natural killer cells (4). These interactions are crucial in determining the immune system’s ability to recognize and eliminate tumor cells. This complex ecosystem offers both therapeutic opportunities and challenges.

## Biomarkers in the tumor-immune landscape

Biomarkers are essential tools in clinical oncology, offering valuable insights into disease progression, therapeutic efficacy, and patient prognosis (5). Within the tumor-immune microenvironment, biomarkers can indicate the status of immune evasion mechanisms, immune infiltration, and tumor antigen expression (6). For instance, immune checkpoint molecules such as PD-1, PD-L1, and CTLA-4 are well-established biomarkers used in immunotherapy, but they do not fully capture the intricate immune evasion strategies employed by tumors (7).

Recent studies have highlighted additional biomarkers, including tumor-associated antigens (TAAs) and tumor-infiltrating lymphocytes (TILs), that are integral to tumor immunity (7). Moreover, the presence of specific cytokines and chemokines in the tumor microenvironment can serve as indicators of immune activation or suppression (8). These biomarkers, along with emerging technologies such as single-cell and bulk RNA sequencing, hold the potential to reveal new therapeutic targets and to guide personalized treatment regimens. In our Research Topic, Yao et al. conducted mRNA sequencing analysis and found that AEG-1 is highly expressed in various cancer types, and is associated with tumor grading and patient prognosis. Additionally, AEG-1 was found to regulate Th1/Th2 immune homeostasis, promote glycogen accumulation, and facilitate tumor fibrosis. This study highlights the potential of AEG-1 as a key biomarker and therapeutic target, offering new insights into its role in tumor progression, immune regulation, and metabolic reprogramming, which could lead to improved prognostic markers and treatment strategies for cancer.

## Mechanisms of immune evasion

Tumor cells employ several strategies to avoid immune detection. One common mechanism involves the downregulation of major histocompatibility complex (MHC) molecules, which are responsible for presenting tumor antigens to immune cells (9). Tumor cells can also secrete immunosuppressive cytokines, such as TGF-β and IL-10, that inhibit the activation of immune effector cells [Wang et al.]. Furthermore, the tumor microenvironment can create physical and metabolic barriers, including hypoxia and acidosis, that hinder immune cell infiltration and function (10).

The concept of immune tolerance is also a central aspect of tumor immunity. Tumor-associated macrophages (TAMs), regulatory T-cells (Tregs), and myeloid-derived suppressor cells (MDSCs) are key players in maintaining immune tolerance within the tumor microenvironment (11). These cells dampen anti-tumor immune responses and support tumor growth. Understanding the molecular mechanisms driving the recruitment and activation of these suppressive cells is crucial for developing strategies to overcome immune resistance.

## Emerging therapies and future directions

The increasing understanding of the tumor-immune interplay has paved the way for novel immunotherapies. Cancer immunotherapies, such as immune checkpoint inhibitors, adoptive T-cell therapy, and cancer vaccines, have shown promising results in clinical trials (12). However, the heterogeneous nature of tumors and their immune microenvironment complicates the development of universal treatments.

Future research will focus on the identification of novel biomarkers that can predict patient response to specific therapies. Moreover, combination therapies that target multiple immune evasion mechanisms are likely to enhance the effectiveness of immunotherapy. Recent advances in personalized medicine and precision oncology offer hope for tailored treatments that take into account individual tumor-immune profiles.

In our Research Topic, Zhu et al. conducted a systematic review and meta-analysis to evaluate the effectiveness and safety of combining PD-1/PD-L1 inhibitors with anti-angiogenic agents in patients with unresectable hepatocellular carcinoma (HCC)364. The analysis included five Phase III randomized controlled trials involving 1515 patients and revealed that combination therapy significantly improved overall survival (OS) and progression-free survival (PFS) compared to monotherapy or standard treatments. Additionally, the combination therapy showed a higher objective response rate (ORR), though it was also associated with a higher risk of adverse events (AEs).The significance of this study lies in its contribution to advancing the treatment options for unresectable HCC, a disease with limited effective therapies. The findings underscore the potential of combining immune checkpoint inhibitors and anti-angiogenic agents as a promising therapeutic strategy, providing a new avenue for improving patient outcomes, while also highlighting the need for careful management of adverse events.

Furthermore, the role of the microbiome in modulating the immune response is an emerging area of interest (13). The gut microbiota, in particular, has been shown to influence the efficacy of immunotherapy, opening new avenues for therapeutic intervention.

## Conclusion

The tumor-immune nexus remains a critical area of research, with vast potential for advancing cancer therapy. As we continue to uncover the molecular and cellular mechanisms that govern tumor-immune interactions, we are moving closer to a future where personalized and targeted immunotherapies can offer more effective treatment options for cancer patients. However, challenges remain, and continued exploration of novel biomarkers, immune evasion mechanisms, and emerging therapies will be essential in shaping the next generation of cancer immunotherapy.

The research presented in this Research Topic highlights the exciting progress being made in this field and provides a glimpse into the future of cancer treatment, where immune-based therapies play a central role in combating this devastating disease.
